# Body mass index at age 18 years and recent body mass index in relation to risk of breast cancer overall and ER/PR/HER2-defined subtypes in white women and African-American women: a pooled analysis

**DOI:** 10.1186/s13058-017-0931-5

**Published:** 2018-01-22

**Authors:** Huiyan Ma, Giske Ursin, Xinxin Xu, Eunjung Lee, Kayo Togawa, Kathleen E. Malone, Polly A. Marchbanks, Jill A. McDonald, Michael S. Simon, Suzanne G. Folger, Yani Lu, Jane Sullivan-Halley, Dennis M. Deapen, Michael F. Press, Leslie Bernstein

**Affiliations:** 1 Department of Population Sciences, Beckman Research Institute, City of Hope, 1500 East Duarte Rd., Duarte, CA 91010 USA; 2 Cancer Registry of Norway, Oslo, Norway; 3 Department of Nutrition, Institute of Basic Medical Sciences, University of Oslo, Oslo, Norway; 4 Department of Preventive Medicine, Keck School of Medicine, University of Southern California, Los Angeles, CA 90033 USA; 5 Section of Environment and Radiation, International Agency for Research on Cancer, Lyon, France; 6 Division of Public Health Sciences, Fred Hutchinson Cancer Research Center, Seattle, WA 98109 USA; 7 Division of Reproductive Health, Centers for Disease Control and Prevention, Atlanta, GA 30333 USA; 8 College of Health and Social Services, New Mexico State University, Las Cruces, NM 88003 USA; 9 Karmanos Cancer Institute, Department of Oncology, Wayne State University, Detroit, MI 48201 USA; 10 Pathology, Keck School of Medicine, University of Southern California, Los Angeles, CA 90033 USA

**Keywords:** Body size, Body mass index, Breast cancer, Triple negative, Luminal-like, African-American women, White women

## Abstract

**Background:**

Although it has been well-documented that obesity is associated with decreased risk of premenopausal breast cancer and increased risk of postmenopausal breast cancer, it is unclear whether these associations differ among breast cancer subtypes defined by the tumor protein expression status of estrogen receptor (ER), progesterone receptor (PR), and human epidermal growth factor receptor 2 (HER2).

**Methods:**

We evaluated the associations of body mass index (BMI) at age 18 years and recent BMI in relation to risk of breast cancer overall and ER/PR/HER2-defined subtypes, in 6320 women (3934 case-patient participants, 2386 control participants) aged 35–64 years, who participated in one of three population-based case-control studies. We estimated multivariable-adjusted odd ratios (ORs) and corresponding 95% confidence intervals (CIs) using polychotomous unconditional logistic regression methods for case-control comparisons in premenopausal women and postmenopausal women.

**Results:**

BMI at age 18 years was inversely associated with risk of breast cancer, particularly among premenopausal women (≥ 25 vs. < 20 kg/m^2^, OR = 0.72, 95% CI = 0.53–0.96; per 5 kg/m^2^ increase, OR = 0.83, 95% CI = 0.73–0.95). This inverse association did not differ across ER/PR/HER2-defined subtypes or by race (white women, African-American women). Recent BMI was not associated with risk of premenopausal breast cancer after adjustment for BMI at age 18 years; nevertheless, the analysis for the joint effects of BMI at age 18 years and recent BMI showed that premenopausal women in the highest categories of the two BMI measures (≥ 25 kg/m^2^ at age 18 years and ≥ 30 kg/m^2^ for recent BMI) had 46% lower risk of breast cancer than premenopausal women in the lowest categories of the two BMI measures (< 20 kg/m^2^ at age 18 years and < 25 kg/m^2^ for recent BMI; OR = 0.54, 95% CI = 0.38–0.78). Neither measure of BMI was statistically significantly associated with risk of postmenopausal breast cancer.

**Conclusion:**

Our findings indicate that high BMI near the end of adolescence decreases risk of all ER/PR/HER2-defined subtypes of premenopausal breast cancer and also suggest that this benefit could be maximized among premenopausal women who consistently have high BMI during their premenopausal years.

## Background

The prevalence of obesity (defined as a body mass index (BMI) of 30 kg/m^2^ or greater) has dramatically increased since the 1980s [1]. Convincing epidemiologic evidence indicates that obesity is associated with decreased risk of premenopausal breast cancer and increased risk of postmenopausal breast cancer [2]. However, it is unclear whether obesity impacts the tumorigenesis of all breast cancers or only certain breast cancer subtypes as defined by the tumor protein expression status of the estrogen receptor (ER), progesterone receptor (PR), and human epidermal growth factor receptor 2 (HER2).

Munsell et al. [3] conducted a meta-analysis of 57 epidemiologic studies published between 1980 and 2012 on the associations between obesity and risk of breast cancer subtypes defined by ER and PR. They reported that obese women had 22% lower risk of premenopausal ER-positive (ER+)/PR+ breast cancer than premenopausal women with BMI lower than 25 kg/m^2^ (summary risk ratio = 0.78, 95% confidence interval (95% CI) = 0.67–0.92) and 39% higher risk of postmenopausal ER+/PR+ breast cancer than postmenopausal women with BMI lower than 25 kg/m^2^ (summary risk ratio = 1.39, 95% CI =1.14–1.70). In the same study, obesity was not associated with the risk of ER-negative (ER–)/PR– breast cancer in either premenopausal women or postmenopausal women.

Pierobon et al. [4] used meta-analysis to summarize the findings of 11 epidemiologic studies published between May 2008 and February 2012 that assessed the association between obesity and risk of triple negative breast cancer (TNBC (ER–/PR–/HER2–)). They concluded that, in a case-case comparison of TNBC or basal-like subtype with receptor-positive subtype, obese premenopausal women had 43% greater risk of TNBC than non-obese premenopausal women, but that obesity was not associated with risk of TNBC in postmenopausal women (premenopausal women, summary odds ratio (OR) = 1.43, 95% CI = 1.23–1.65; postmenopausal women, summary OR = 0.99, 95% CI = 0.79–1.24).

Bandera et al. [5] present results from the African American Breast Cancer Epidemiology and Risk (AMBER) Consortium showing that, among premenopausal women, BMI at age 18 years was inversely associated with risk of ER+ breast cancer but not with risk of ER– breast cancer or TNBC (BMI ≥30 vs. BMI = 20–24.9 kg/m^2^, ER+ OR = 0.65, 95% CI = 0.42–1.01; ER– OR = 1.00, 95% CI = 0.63–1.58; TNBC OR = 1.08, 95% CI = 0.59–1.98). However, among postmenopausal women, BMI at age 18 years was associated with decreased risk of all subtypes of breast cancer (BMI ≥30 vs. BMI = 20–24.9 kg/m^2^, ER+ OR = 0.62, 95% CI = 0.38–1.01; ER– OR = 0.78, 95% CI = 0.44–1.41; TNBC OR = 0.68, 95% CI = 0.29–1.56). They also found that high BMI immediately before diagnosis (cases) or an index date (controls) was not associated with the risk of ER–, ER+, or TNBC in premenopausal women, but was associated with 31% increased risk of ER+ breast cancer (BMI ≥35 vs. BMI < 25 kg/m^2^, OR =1.31, 95% CI = 1.02–1.67) and 40% decreased risk of TNBC in postmenopausal women (BMI ≥ 35 vs. BMI < 25 kg/m^2^, OR =0.60, 95% CI = 0.39–0.93).

Given the lack of consistency in results for analysis of BMI in relation to tumor marker subtypes of breast cancer, especially TNBC, additional research is needed to clarify whether obesity impacts all subtypes of breast cancer or only certain receptor-defined subtypes. Moreover, it remains unclear whether associations differ among racial/ethnic groups.

Here we present results from a pooled analysis of data from three population-based breast cancer case-control studies conducted among white women and African-American women [6–8]; we examine the associations between BMI at age 18 years and BMI 5 years before case patients’ breast cancer diagnosis or control participants’ index date (herein referred to as “recent BMI”) in relation to risk of breast cancer overall and risk of breast cancer subtypes defined by ER, PR, and HER2.

## Methods

### Study population and data collection

Eligible participants for this analysis were women who participated in one of the three population-based case-control studies – the Women’s Contraceptive and Reproductive Experiences (CARE) Study [6], the Women’s Breast Carcinoma in situ (BCIS) Study [7], or the Women’s Learning the Influence of Family and Environment (LIFE) Study [8].

The Women’s CARE Study was a population-based, multi-site, case-control study designed to examine risk factors for invasive breast cancer among US-born white women and African-American women [9]. The age distribution and participant response rates by study site, case-control status, and race have previously been published [9]. The Women’s CARE Study selected a stratified (by age group) random sample of women aged 35 to 64 years who were newly diagnosed with their first primary, histologically confirmed invasive breast cancer (International Classification of Diseases for Oncology (ICD-O) codes C50.0–C50.9) between July 1994 and April 1998. African-American women were oversampled to maximize their numbers in the study, and white women were sampled to provide approximately equal numbers of women in each 5-year age category (from 35 to 64 years). Control participants were women with no history of invasive or in situ breast cancer who were identified by random digit dialing between August 1994 and December 1998; control participants were frequency matched to the expected age and race distribution of cases within each geographic region of residence [6].

The Women’s CARE Study participants included in this pooled analysis are from Los Angeles and Detroit, the two study sites where tumor tissue samples were collected. Details of tissue collection for case-patient participants at the two sites have been described previously [6]. The Women’s CARE Study recruited 1921 case-patient participants (1072 white and 849 African-American women) and 2034 control participants (1161 white and 873 African-American women) from Los Angeles and Detroit. Of 1921 case-patient participants, 1206 had ER/PR/HER2 status assessed in a centralized pathology laboratory at the University of Southern California (USC).

The Women’s BCIS Study investigated risk factors for BCIS among US-born white women and African-American women who resided in Los Angeles County [7]. Case-patient participants were US-born and English speaking white women and African-American women ages 35–64 years, who were newly diagnosed with a first primary BCIS (ICD-O codes C50.0–C50.9) between March 1995 and April 1998 (n = 567; 475 white and 92 African-American women). The Women’s BCIS Study was conducted at the same time as the Women’s CARE Study and used the same methodology. Specifically, the two studies used the same questionnaire, same study interviewers, and same central laboratory and classification scheme for ER, PR, and HER2 status (see below). In addition, Los Angeles control participants from the Women’s CARE Study also served as controls for the BCIS Study. For this pooling project, we excluded 37 Women’s BCIS Study case-patient participants with lobular carcinoma in situ (LCIS, ICD-O morphology code 8520) because LCIS is generally not included in clinical definitions of in situ breast cancer [10]; thus 530 case-patient participants (444 white and 86 African-American women) were considered eligible for this pooling project. Among these case patients, 343 had ER/PR/HER2 status assessed by the centralized pathology laboratory at USC.

The Women’s LIFE Study investigated genetic and epidemiologic risk factors for invasive breast cancer in US-born white women and African-American women who resided in Los Angeles County [8, 11]. Case-patient participants were women aged 20–49 years who were diagnosed with a first primary invasive breast cancer (ICD-O codes: C50.0–C50.9) between February 1998 and May 2003 (n = 1794; 1585 white and 209 African-American women). Control participants were women ages 20–49 years who had no history of invasive or in situ breast cancer. Control participants were individually matched by race (white and African-American), age (within 5 years and ages 20–49 years), and neighborhood of residence, to the subset of case-patient participants who were diagnosed between 1 July 2000 and 31 May 2003 (n = 444; 409 white and 35 African-American women). The Women’s LIFE Study used an expanded version of the Women’s CARE Study questionnaire and abstracted tumor marker status from pathology reports collected by the Los Angeles Cancer County Surveillance Program (LACSP) with ER available for 1569 (87.4%) patients, PR available for 1439 (80.2%) patients, and HER2 available for 1206 (67.3%) patients.

For all three studies, detailed information about body size measures and covariates prior to the reference date was collected by trained staff in standardized in-person interviews. The reference date for a case-patient participant was the date of her breast cancer diagnosis; the reference date for a control participant was the date she was identified by random digit dialing in the Women’s CARE Study, or the date of initial contact in the Women’s LIFE Study.

Anthropometric variables involved in this analysis include: tallest height without shoes, usual weight (if pregnant, the pre-pregnancy weight was used) at age 18 years and 5 years before reference age (termed “recent weight”). BMI at each time point was calculated as the corresponding body weight in kilograms divided by height in meters squared (kg/m^2^). Based on the World Health Organization’s guideline [12], three categories of “recent BMI” were created: underweight/normal weight, <25.0 kg/m^2^; overweight, 25.0–29.9 kg/m^2^; and obese, ≥30.0 kg/m^2^. Because only 1.6% of women at age 18 years had a BMI of 30 kg/m^2^ or higher and 47.8% had a BMI that was lower than 20.0 kg/m^2^, we used previously published categories for BMI at age 18 years (< 20.0, 20.0–24.9, ≥25.0 kg/m^2^) [13]. None of our three source studies has published on the association between BMI and ER/PR/HER2-defined breast cancer subtypes, whereas two of our three source studies previously published papers describing the association between BMI and ER/PR-defined subtypes [8, 14].

After pooling the data from the three source studies, 6723 women (4245 case-patient participants and 2478 control participants) were potentially eligible for this analysis. We excluded 160 case-patient participants and 64 control participants for whom information was missing on the following factors: age at menarche (4 cases, 1 control), parity (8 cases, 6 controls), duration of oral contraceptive use (23 cases, 5 controls), education (15 cases, 1 control), BMI at age 18 years (15 cases, 4 controls), recent BMI (18 cases, 12 controls), recreational physical activity (6 cases, 3 controls), smoking status (8 cases), alcohol intake (10 cases, 2 controls), and menopausal status (53 cases, 30 controls). We were unable to determine menopausal status for 53 case patients and 30 control participants who had a hysterectomy with at least a portion of one ovary remaining (9 cases, 12 controls), began menopausal hormone therapy (MHT) use within 12 months of their last menstrual period (20 cases, 10 controls), or did not answer the questions regarding menopausal status and MHT use (24 cases, 8 controls). We also excluded 179 participants (151 cases, 28 controls) in the Women’s LIFE Study who were younger than 35 years at diagnosis or reference date, since the age range for the Women’s CARE and BCIS studies was 35 to 64 years.

After these exclusions, 3934 case-patient participants (1873 from the Women’s CARE Study, 517 from the Women’s BCIS Study, and 1544 from the Women’s LIFE Study) and 2386 control participants (1982 from the Women’s CARE Study, of whom 1226 Los Angeles control participants were also used for the Women’s BCIS Study, and 404 from the Women’s LIFE Study) remained and were included in the pooled analysis.

### Assessment of biomarkers

As noted above, we determined ER/PR/HER2 receptor status of the breast cancers in case-patient participants in the Women’s CARE Study and Women’s BCIS Study in a centralized clinical laboratory improvement act (CLIA)-approved, College of American Pathologists (CAP)-certified pathology laboratory at USC using immunohistochemistry (IHC) methods [15, 16]. For ER and PR, at least 100 tumor cells were examined for each specimen; a specimen was considered as positive for the receptor if at least 1% of the tumor cell nuclei were immunostained [17]. HER2 expression was determined by IHC using the 10H8 monoclonal antibody [18, 19]. No (0) or weak (1+) membrane immunostaining was considered HER2–. Moderate (2+) or strong membrane immunostaining (3+) was considered HER2+, based on previous validation results from the same pathology laboratory [18]. ER/PR/HER2 status for case-patient participants in the Women’s LIFE Study was abstracted from pathology reports collected through the LACSP [20], a member of the population-based California Cancer Registry and also sponsored by the National Cancer Institute’s Surveillance, Epidemiology and End Results (SEER) program.

Of 3934 case-patient participants, 2861 (72.7%) had ER status, 2740 (69.7%) had PR status, and 2560 (65.1%) had HER2 status. In our analysis, we classified case-patient participants into four subgroups: TNBC (ER–/PR–/HER2–, *n* = 515), luminal-like breast cancer (ER+ and/or PR+, *n* = 2056), HER2-enriched breast cancer (ER–/PR–/HER2+, n = 212), and an unclassified group (*n* = 1151) [21]. To determine whether the impact of BMI varied across subtypes of luminal-like breast cancer, we further classified 1797 luminal-like tumors with information available for all three markers into luminal A-like (ER+/PR+/HER2–, *n* = 1175), luminal B-like-HER2– (ER+ or PR+ plus HER2–, *n* = 283), or luminal B-like-HER2+ (ER+ and/or PR+ plus HER2+, *n* = 339), based on the 13th St. Gallen International Breast Cancer Conference (2013) Expert Panel recommendation [22]. It is noteworthy that the St. Gallen Panel recommendation requires information on Ki-67 and percentage of PR in PR+ tumors; however, we lacked data on Ki-67 in each of the studies and did not have quantitative data for PR in the Women’s LIFE Study.

### Statistical analyses

We assessed whether BMI at age 18 years and recent BMI were associated with breast cancer overall and ER/PR/HER2-defined subtypes, estimating ORs and corresponding 95% CIs from multivariable polychotomous unconditional logistic regression models [23]. These models were fit separately for premenopausal women and postmenopausal women. For each of the BMI measures, we first estimated the OR and 95% CI associated with each level of a categorical variable. We then estimated the OR and 95% CI associated with each 5 kg/m^2^ increase in the BMI measure, and assessed whether this 5 kg/m^2^ increase in BMI differed from the null using the Wald chi-square test. Finally after conducting these analyses for each subtype, we tested for homogeneity of the 5 kg/m^2^ slope coefficients across ER/PR/HER2-defined subtypes.

In our analysis, women were considered premenopausal if they were still menstruating and had not taken any MHT during the 12 months before the reference date. We classified women as postmenopausal if they had experienced a natural menopause (had a final menstrual period > 12 months before the reference date and had not used MHT before or during the 12-month interval after the last menstrual period), had a surgical menopause (had undergone bilateral oophorectomy with the second ovary removed at least 12 months before the reference date), or had an induced menopause (periods stopped because of chemotherapy or radiation therapy at least 12 months before the reference date). Considering the possibility that associations with recent BMI among postmenopausal women might be modified by MHT use [24], we also assessed these associations stratified by MHT use.

For premenopausal women, in which we observed an inverse association between BMI at age 18 years and breast cancer risk, we further conducted race-stratified analyses (white women, African-American women) for breast cancer overall and for two major subtypes (luminal-like and TNBC). Finally, we assessed the joint effects of BMI at age 18 years and recent BMI (using a variable that combined these two variables) in premenopausal women for breast cancer overall and for the luminal-like subtype (the most common subtype). We did not do race-stratified analyses for the HER2-enriched subtype nor did we assess the joint effects of BMI at age 18 years and recent BMI for either the HER2-enriched subtype or TNBC, due to limited sample size. For postmenopausal women, we did not conduct race-stratified analyses or assess the joint effects of the two BMI measures due to the limited numbers of postmenopausal women in some subgroups.

Our models assessing effects of BMI at age 18 years or recent BMI included both BMI variables (i.e., each was mutually adjusted for the other). All models also included the following factors, selected a priori, as potential confounders in all multivariable models: source study (the Women’s CARE Study or the Women’s BCIS Study, the Women’s LIFE Study), study site (Los Angeles, Detroit), race (white, African-American), education as a proxy for socioeconomic status (high school or lower level of education, technical school or some college, college graduate), reference age (premenopausal women < 40, 40–44, ≥ 45 years; postmenopausal women < 50, 50–54, 55–59, 60–64 years), family history of breast cancer (first-degree (mother, sister, or daughter), no first-degree family history), age at menarche (≤ 12, 13, ≥ 14 years), number of completed (greater than 26-week gestation) pregnancies (never pregnant, 1, 2, ≥ 3, only non-completed pregnancy), lifetime recreational physical activity (inactive, ≤ 2.2, 2.3–6.6, 6.7–15.1, ≥15.2 annual metabolic equivalents of energy expenditure (MET) hour/week), alcohol intake (never, former, current), cigarette smoking status (never, former, current), and oral contraceptive use (never, < 1, 1–4, 5–9, ≥ 10 years). In the analyses of postmenopausal women overall, we additionally adjusted for MHT use (never use, ever use).

We repeated our analyses limiting data to the two source studies with ER/PR/HER2 status obtained by the centralized laboratory to assess whether using ER/PR/HER2 status from multiple laboratories in the LIFE Study (obtained from the LACSP) would influence our results. In another analysis, we restricted case-patient participant data to those obtained from women diagnosed with invasive breast cancer.

In reporting the results of statistical tests determining whether the 5 kg/m^2^ increase in BMI (slope coefficient) differed from the null (test for trend) or whether slope coefficients differed across ER/PR/HER2-defined subtypes (test for homogeneity of trends) we considered a two-sided *P* value less than 0.05 as statistically significant. All analyses were performed using the SAS statistical package (Version 9.4, SAS Institute, Cary, NC, USA).

## Results

### Characteristics of cases and controls

Overall, mean age at reference date was 47.3 and 47.9 years for case-patient participants and control participants, respectively (Table 1). By menopausal status, 61.7% of case-patient participants were premenopausal and 38.3% were postmenopausal; among control participants, 52.7% were premenopausal and 47.3% were postmenopausal. By race, 72.4% of case-patient participants were white women and 27.6% were African-American women; among control participants, 63.0% were white women and 37.0% were African-American women. Overall, the percentage of participants who were overweight or obese during early adulthood was 6.9% and 9.0% for case-patient and control participants, respectively; the percentage of recent obesity was 17.1% and 19.4% for case-patient and control participants, respectively.

**Table 1:** Characteristics of breast cancer case-patient participants and control participants by study

	Overall		Women’s CARE Study		Women’s BCIS Study	Women’s LIFE Study	
	Number of cases (%)	Number of controls (%)	Number of cases (%)	Number of controls^a^ (%)	Number of cases (%)	Number of cases (%)	Number of controls (%)
Number of participants	3934	2386	1873	1982	517	1544	404
Subtype of breast cancer							
Unclassified	1151	─	691	─	182	278	─
Classified	2783	─	1182	─	335	1266	─
Triple negative (ER–/PR–/HER2–)	515 (18.5)	─	329 (27.8)	─	21 (6.3)	165 (13.0)	─
Luminal-like (ER+ and/or PR+)	2056 (73.9)		758 (64.1)		277 (82.7)	1021 (80.7)	
HER2-enriched (ER–/PR–/HER2+)	212 (7.6)	─	95 (8.0)	─	37 (11.0)	80 (6.3)	─
Mean age at reference date, years (SD)	47.3 (7.4)	47.9 (8.1)	48.9 (8.5)	48.8 (8.4)	51.5 (7.3)	44.0 (3.9)	43.5 (3.8)
Menopausal status							
Premenopausal	2427 (61.7)	1257 (52.7)	929 (49.6)	927 (46.8)	203 (39.3)	1295 (83.9)	330 (81.7)
Postmenopausal	1507 (38.3)	1129 (47.3)	944 (50.4)	1055 (53.2)	314 (60.7)	249 (16.1)	74 (18.3)
Never used MHT	650 (43.1)	466 (41.3)	409 (43.3)	430 (40.8)	107 (34.1)	134 (53.8)	36 (48.7)
Ever used MHT	857 (56.9)	663 (58.7)	535 (56.7)	625 (59.2)	207 (65.9)	115 (46.2)	38 (51.3)
Race							
White	2849 (72.4)	1503 (63.0)	1056 (56.4)	1134 (57.2)	435 (84.1)	1358 (88.0)	369 (91.3)
African-American	1085 (27.6)	883 (37.0)	817 (43.6)	848 (42.8)	82 (15.9)	186 (12.0)	35 (8.7)
BMI at age 18 years, kg/m^2^							
< 20.0	1921 (48.8)	1110 (46.5)	882 (47.1)	906 (45.7)	286 (55.3)	753 (48.8)	204 (50.5)
20.0–24.9	1740 (44.2)	1061 (44.5)	848 (45.3)	893 (45.1)	207 (40.0)	685 (44.4)	168 (41.6)
≥ 25.0	273 (6.9)	215 (9.0)	143 (7.6)	183 (9.2)	24 (4.6)	106 (6.9)	32 (7.9)
Recent BMI, kg/m^2^							
< 25.0	2292 (58.3)	1271 (53.3)	985 (52.6)	1003 (50.6)	345 (66.7)	962 (62.3)	268 (66.3)
25.0–29.9	968 (24.6)	652 (27.3)	511 (27.3)	570 (28.8)	115 (22.2)	342 (22.2)	82 (20.3)
≥ 30.0	674 (17.1)	463 (19.4)	377 (20.1)	409 (20.6)	57 (11.0)	240 (15.5)	54 (13.4)

*Abbreviations*: *ER* estrogen receptor, *PR* progesterone receptor, *HER2*, human epidermal growth factor receptor-2, *BMI* body mass index, *CARE* Contraceptive and Reproductive Experiences, *BCIS* Breast Carcinoma in situ, *LIFE* Learning the Influence of Family and Environment, *MHT* menopausal hormone therapy

^a^Including those who also served as controls for the Women’s BCIS Study

### Risk of breast cancer overall and ER/PR/HER2-defined subtypes associated with BMI at age 18 years or recent BMI

In premenopausal women, BMI at age 18 years was inversely associated with risk of breast cancer overall (≥ 25 vs. < 20 kg/m^2^, OR = 0.72, 95% CI = 0.53–0.96; per 5 kg/m^2^ increase, OR = 0.83, 95% CI = 0.73–0.95, Table 2). The inverse association per 5 kg/m^2^ increase in BMI at age 18 years did not differ by subtype (*P* for homogeneity of slope coefficients per 5 kg/m^2^ increase = 0.15).

**Table 2:** Adjusted^a^ odds ratio (OR) and 95% confidence interval (CI) for breast cancer overall and ER/PR/HER2-defined subtypes associated with BMI in premenopausal women and postmenopausal women

	Controls	All cases		Triple negative (ER–/PR–/HER2–)		Luminal-like (ER+ and/or PR+)		HER2-enriched (ER–/PR–/HER2+)		Unclassified	
	Number	Number	OR (95% CI)	Number	OR (95% CI)	Number	OR (95% CI)	Number	OR (95% CI)	Number	OR (95% CI)
Premenopausal											
BMI at age 18 years, kg/m^2^											
< 20.0	588	1187	Referent	160	Referent	656	Referent	71	Referent	300	Referent
20.0–24.9	543	1067	0.99 (0.84–1.16)	144	0.91 (0.69–1.20)	577	0.97 (0.81–1.17)	55	0.82 (0.55–1.22)	291	1.09 (0.88–1.35)
≥ 25.0	126	173	0.72 (0.53–0.96)	25	0.62 (0.36–1.04)	83	0.63 (0.44–0.91)	6	0.36 (0.14–0.92)	59	1.00 (0.68–1.46)
Per 5 kg/m^2^ increase			0.83 (0.73–0.95)		0.68 (0.53–0.87)		0.80 (0.68–0.95)		0.59 (0.40–0.86)		1.00 (0.84–1.19)
*P* value			0.007		0.002		0.008		0.006		0.98
					*P* value for homogeneity of slope coefficients across three subtypes = 0.15						
Recent BMI, kg/m^2^											
< 25.0	758	1545	Referent	194	Referent	868	Referent	80	Referent	403	Referent
25.0–29.9	299	533	0.90 (0.74–1.08)	80	1.07 (0.78–1.47)	269	0.82 (0.65–1.02)	29	0.99 (0.61–1.60)	155	0.92 (0.71–1.17)
≥ 30.0	200	349	0.85 (0.67–1.08)	55	1.12 (0.75–1.67)	179	0.80 (0.60–1.06)	23	1.34 (0.75–2.38)	92	0.75 (0.54–1.03)
Per 5 kg/m^2^ increase			1.00 (0.92–1.08)		1.14 (1.00–1.31)		0.97 (0.88–1.07)		1.19 (0.98–1.45)		0.94 (0.84–1.05)
*P* value			0.94		0.06		0.58		0.07		0.26
					*P* value for homogeneity of slope coefficients across three subtypes = 0.02						
Postmenopausal^b^											
BMI at age 18 years, kg/m^2^											
< 20.0	522	734	Referent	89	Referent	358	Referent	40	Referent	247	Referent
20.0–24.9	518	673	0.92 (0.78–1.10)	83	0.97 (0.69–1.39)	326	0.94 (0.76–1.17)	35	0.88 (0.53–1.44)	229	0.89 (0.70–1.12)
≥ 25.0	89	100	0.79 (0.56–1.11)	14	1.01 (0.52–1.98)	56	0.99 (0.65–1.50)	5	0.76 (0.27–2.14)	25	0.51 (0.31–0.85)
Per 5 kg/m^2^ increase			0.98 (0.85–1.14)		0.98 (0.73–1.32)		1.12 (0.94–1.35)		0.76 (0.48–1.20)		0.86 (0.70–1.05)
*P* value			0.81		0.89		0.21		0.23		0.14
					*P* value for homogeneity of slope coefficients across three subtypes = 0.20						
Recent BMI, kg/m^2^											
< 25.0	513	747	Referent	83	Referent	390	Referent	36	Referent	238	Referent
25.0–29.9	353	435	0.95 (0.78–1.16)	64	1.11 (0.76–1.63)	193	0.88 (0.69–1.12)	28	1.23 (0.71–2.12)	150	0.93 (0.71–1.21)
≥ 30.0	263	325	1.09 (0.86–1.39)	39	0.95 (0.59–1.55)	157	1.19 (0.89–1.60)	16	1.11 (0.55–2.25)	113	1.03 (0.75–1.41)
Per 5 kg/m^2^ increase			0.97 (0.90–1.06)		0.97 (0.82–1.15)		0.98 (0.89–1.09)		1.06 (0.83–1.34)		0.95 (0.85–1.07)
*P* value			0.54		0.74		0.74		0.66		0.39
					*P* value for homogeneity of slope coefficients across three subtypes = 0.83						
Postmenopausal/never used MHT											
Recent BMI, kg/m^2^											
< 25.0	186	283	Referent	33	Referent	148	Referent	15	Referent	87	Referent
25.0–29.9	150	181	0.94 (0.68–1.29)	27	0.92 (0.50–1.71)	74	0.82 (0.55–1.21)	11	0.98 (0.41–2.34)	69	1.06 (0.70–1.61)
≥ 30.0	130	186	1.39 (0.98–1.99)	20	0.96 (0.50–2.00)	86	1.55 (1.00–2.41)	9	1.20 (0.44–3.30)	71	1.42 (0.90–2.25)
Per 5 kg/m^2^ increase			1.07 (0.95–1.21)		0.99 (0.77–1.27)		1.07 (0.92–1.25)		1.10 (0.77–1.56)		1.10 (0.94–1.28)
*P* value			0.27		0.94		0.36		0.60		0.26
					*P* value for homogeneity of slope coefficients across three subtypes = 0.81						
Postmenopausal/ever used MHT											
Recent BMI, kg/m^2^											
< 25.0	327	464	Referent	50	Referent	242	Referent	21	Referent	151	Referent
25.0–29.9	203	254	0.93 (0.72–1.21)	37	1.12 (0.67–1.87)	119	0.89 (0.65–1.22)	17	1.41 (0.68–2.90)	81	0.86 (0.60–1.22)
≥ 30.0	133	139	0.86 (0.62–1.20)	19	0.87 (0.44–1.69)	71	0.94 (0.63–1.42)	7	0.91 (0.33–2.50)	42	0.75 (0.47–1.19)
Per 5 kg/m^2^ increase			0.90 (0.79–1.01)		0.96 (0.75–1.21)		0.92 (0.79–1.06)		1.01 (0.71–1.42)		0.82 (0.69–0.98)
*P* value			0.07		0.71		0.24		0.98		0.03
					*P* value for homogeneity of slope coefficients across three subtypes = 0.85						

*Abbreviations*: *ER* estrogen receptor, *PR* progesterone receptor, *HER2* human epidermal growth factor receptor-2, *BMI* body mass index, *MHT* menopausal hormone therapy

^a^ORs obtained from multivariable polychotomous unconditional logistic regression models with mutual adjustment of BMI at age 18 years and recent BMI and with adjustment for source study, study site, race, education, reference age, first-degree breast cancer family history, age at menarche, number of completed pregnancies, lifetime recreational physical activity, alcohol intake, cigarette smoking status, and oral contraceptive use

^b^Model described (^a^) is additionally adjusted for MHT use

Recent BMI was not associated with risk of premenopausal breast cancer overall. However, our analyses of ER/PR/HER2-defined subtypes in premenopausal women showed some evidence that recent obesity affected ER/PR/HER2-defined subtypes differently, with no association for luminal-like subtype and potentially increased risks of TNBC and HER2-enriched subtypes (per 5 kg/m^2^ increase, luminal-like OR = 0.97, 95% CI = 0.88–1.07; TNBC OR = 1.14, 95% CI = 1.00–1.31; HER2-enriched OR = 1.19, 95% CI = 0.98–1.45; *P* for homogeneity of slope coefficients per 5 kg/m^2^ increase = 0.02).

In postmenopausal women, BMI at age 18 years was not associated with risk of breast cancer overall although the OR was reduced more than 20% when comparing overweight and obese women to thin women (≥ 25 vs. < 20 kg/m^2^, OR = 0.79, 95% CI = 0.56–1.11; per 5 kg/m^2^ increase, OR = 0.98, 95% CI = 0.85–1.14); no differences in risk across ER/PR/HER2-defined subtypes was observed (*P* value for homogeneity of regression coefficients = 0.20). Furthermore, recent BMI was not associated with risk of breast cancer overall, TNBC, luminal-like subtype, or HER2-enriched subtype in postmenopausal women. Among postmenopausal women who had never used MHT, risk of the luminal-like subtype was 1.5-fold greater among women who were obese than among women who were normal weight or thin (≥ 30 vs. < 25 kg/m^2^, OR = 1.55, 95% CI = 1.00–2.41).

Analyses sub-classifying luminal-like breast cancer into luminal A-like, luminal B-like-HER2–, and luminal B-like-HER2+, did not provide any evidence that the associations of BMI at age 18 years or recent BMI in either premenopausal women or postmenopausal women varied across these breast cancer subtypes (results not shown).

### Race-stratified risk of premenopausal breast cancer overall, TNBC, and luminal-like subtype associated with BMI at age 18 years or recent BMI

A modest inverse association was observed between BMI at age 18 years and premenopausal breast cancer overall in both white women and African-American women (per 5 kg/m^2^ increase, white women, OR = 0.86, 95% CI = 0.73–1.02; African-American women, OR = 0.79, 95% CI = 0.62–1.00, Table 3). The inverse associations were also observed with two major ER/PR/HER2-defined subtypes (luminal-like and TNBC) in both premenopausal white women (per 5 kg/m^2^ increase, OR for TNBC = 0.61, 95% CI = 0.44–0.86; OR for luminal-like subtype = 0.87, 95% CI = 0.71–1.06) and premenopausal African-American women (per 5 kg/m^2^ increase, OR for TNBC = 0.78, 95% CI = 0.54–1.15; OR for luminal-like subtype = 0.74, 95% CI = 0.54–1.03).

**Table 3:** Adjusted^a^ odds ratio (OR) and 95% confidence interval (CI) for breast cancer overall, triple negative breast cancer, and luminal-like subtype associated with BMI in premenopausal white women and premenopausal African-American women

	All cases		Triple negative (ER–/PR–/HER2–)		Luminal-like (ER+ and/or PR+)	
	Number	OR (95% CI)	Number	OR (95% CI)	Number	OR (95% CI)
Premenopausal white women						
BMI at age 18 years, kg/m^2^						
< 20.0	946	Referent	108	Referent	553	Referent
20.0–24.9	793	1.01 (0.84–1.22)	91	0.92 (0.66–1.30)	470	1.03 (0.83–1.28)
≥ 25.0	121	0.76 (0.53–1.10)	12	0.53 (0.26–1.09)	64	0.73 (0.47–1.13)
Per 5 kg/m^2^ increase		0.86 (0.73–1.02)		0.61 (0.44–0.86)		0.87 (0.71–1.06)
*P* value		0.08		0.004		0.16
			*P* value for homogeneity of slope coefficients between two subtypes = 0.04			
Recent BMI, kg/m^2^						
< 25.0	1274	Referent	136	Referent	754	Referent
25.0–29.9	373	0.87 (0.69–1.10)	51	1.30 (0.87–1.93)	205	0.78 (0.59–1.02)
≥ 30.0	213	0.72 (0.53–0.97)	24	1.00 (0.57–1.75)	128	0.71 (0.50–1.01)
Per 5 kg/m^2^ increase		0.95 (0.86–1.05)		1.12 (0.94–1.34)		0.94 (0.83–1.06)
*P* value		0.32		0.19		0.28
			*P* value for homogeneity of slope coefficients between two subtypes = 0.04			
Premenopausal African-American women						
BMI at age 18 years, kg/m^2^						
< 20.0	241	Referent	52	Referent	103	Referent
20.0–24.9	274	0.98 (0.72–1.33)	53	0.92 (0.56–1.50)	107	0.90 (0.61–1.35)
≥ 25.0	52	0.66 (0.40–1.13)	13	0.71 (0.31–1.65)	19	0.60 (0.29–1.24)
Per 5 kg/m^2^ increase		0.79 (0.62–1.00)		0.78 (0.54–1.15)		0.74 (0.54–1.03)
*P* value		0.05		0.21		0.07
			*P* value for homogeneity of slope coefficients between two subtypes = 0.80			
Recent BMI, kg/m^2^						
< 25.0	271	Referent	58	Referent	114	Referent
25.0–29.9	160	0.92 (0.66–1.29)	29	0.79 (0.45–1.37)	64	0.93 (0.60–1.44)
≥ 30.0	136	1.02 (0.68–1.53)	31	1.09 (0.58– 2.05)	51	0.94 (0.55–1.59)
Per 5 kg/m^2^ increase		1.08 (0.92–1.25)		1.13 (0.90–1.43)		1.04 (0.85–1.28)
*P* value		0.35		0.30		0.69
			*P* value for homogeneity of slope coefficients between two subtypes = 0.53			

*Abbreviations*: *ER* estrogen receptor, *PR* progesterone receptor, *HER2*, human epidermal growth factor receptor-2, *BMI* body mass index

^a^ORs obtained from multivariable polychotomous unconditional logistic regression models with mutual adjustment of BMI at age 18 years and recent BMI and with adjustment for source study, study site, education, reference age, first-degree breast cancer family history, age at menarche, number of completed pregnancies, lifetime recreational physical activity, alcohol intake, cigarette smoking status, and oral contraceptive use

In premenopausal white women, recent obesity was associated with a reduced risk for breast cancer overall and for luminal-like subtype, but was not associated with TNBC (≥ 30 vs. < 25 kg/m^2^, overall OR = 0.72, 95% CI = 0.53–0.97; luminal-like OR = 0.71, 95% CI = 0.50–1.01; TNBC OR = 1.00, 95% CI = 0.57–1.75). Recent BMI was not associated with risk of breast cancer overall or the two subtypes in premenopausal African-American women (≥ 30 vs. < 25 kg/m^2^, overall OR = 1.02, 95% CI = 0.68–1.53; luminal-like OR = 0.94, 95% CI = 0.55–1.59; TNBC OR = 1.09, 95% CI = 0.58–2.05).

### Joint effect of BMI at age 18 years and recent BMI on risk of premenopausal breast cancer overall and risk of luminal-like breast cancer

Premenopausal women in the highest categories of the two BMI measures (≥ 25 kg/m^2^ at age 18 years and ≥ 30 kg/m^2^ for recent BMI) had 46% lower risk of breast cancer overall (OR = 0.54 and 95% CI = 0.38–0.78, Table 4) and 54% lower risk of luminal-like subtype (OR = 0.46 and 95% CI = 0.29–0.73) than premenopausal women whose BMI was in the lowest categories for both measures (< 20 kg/m^2^ for BMI at age 18 years and < 25 kg/m^2^ for recent BMI).

**Table 4:** Adjusted^a^ odds ratio (OR) and 95% confidence interval (CI) for joint effect of BMI at age 18 years and recent BMI in relation to risk of breast cancer overall and luminal-like subtype in premenopausal women

BMI at age 18 years, kg/m^2^	Recent BMI, kg/m^2^	Controls	All cases		luminal-like (ER+ and/or PR+)	
		Number	Number	OR (95% CI)	Number	OR (95% CI)
Overall						
< 20.0	< 25.0	472	952	Referent	540	Referent
< 20.0	25.0–29.9	96	184	0.93 (0.70–1.24)	92	0.79 (0.56–1.13)
< 20.0	≥ 30.0	20	51	1.21 (0.69–2.13)	24	0.99 (0.50–1.96)
20.0–24.9	< 25.0	265	555	1.03 (0.85–1.25)	311	1.00 (0.79–1.26)
20.0–24.9	25.0–29.9	171	302	0.87 (0.69–1.10)	151	0.77 (0.58–1.02)
20.0–24.9	≥ 30.0	107	210	0.86 (0.65–1.13)	115	0.84 (0.60–1.17)
≥ 25.0	< 25.0	21	38	0.89 (0.50–1.58)	17	0.65 (0.32–1.35)
≥ 25.0	25.0–29.9	32	47	0.75 (0.46–1.22)	26	0.79 (0.43–1.44)
≥ 25.0	≥ 30.0	73	88	0.54 (0.38–0.78)	40	0.46 (0.29–0.73)
Restricted to studies in which cases’ ER/PR/HER2 status determined in a central laboratory						
< 20.0	< 25.0	342	448	Referent	183	Referent
< 20.0	25.0–29.9	70	86	0.94 (0.66–1.35)	31	0.79 (0.49–1.29)
<20.0	≥ 30.0	16	21	1.03 (0.52–2.05)	6	0.67 (0.25–1.83)
20.0–24.9	< 25.0	186	253	1.05 (0.83–1.34)	103	1.03 (0.75–1.42)
20.0–24.9	25.0–29.9	132	148	0.86 (0.65–1.15)	55	0.75 (0.51–1.11)
20.0–24.9	≥ 30.0	81	89	0.78 (0.55–1.11)	37	0.79 (0.50–1.25)
≥ 25.0	< 25.0	15	23	1.09 (0.55–2.16)	8	0.83 (0.33–2.08)
≥ 25.0	25.0–29.9	27	23	0.68 (0.38–1.22)	11	0.76 (0.36–1.62)
≥ 25.0	≥ 30.0	58	41	0.52 (0.33–0.81)	15	0.46 (0.25–0.86)
Restricted to invasive cases						
< 20.0	< 25.0	472	849	Referent	485	Referent
< 20.0	25.0–29.9	96	169	0.94 (0.70–1.26)	86	0.83 (0.58–1.20)
< 20.0	≥ 30.0	20	51	1.35 (0.77–2.38)	24	1.13 (0.56–2.28)
20.0–24.9	< 25.0	265	506	1.04 (0.85–1.27)	287	1.03 (0.81–1.32)
20.0–24.9	25.0–29.9	171	286	0.92 (0.72–1.17)	143	0.84 (0.62–1.13)
20.0–24.9	≥ 30.0	107	201	0.89 (0.67–1.18)	114	0.94 (0.67–1.34)
≥ 25.0	< 25.0	21	33	0.87 (0.48–1.59)	14	0.58 (0.27–1.26)
≥ 25.0	25.0–29.9	32	44	0.80 (0.48–1.32)	25	0.89 (0.48–1.66)
≥ 25.0	≥ 30.0	73	85	0.58 (0.40–0.84)	40	0.53 (0.33–0.85)

*Abbreviations*: *ER* estrogen receptor, *PR* progesterone receptor, *HER2* human epidermal growth factor receptor-2, *BM*I body mass index

^a^ORs obtained from multivariable polychotomous unconditional logistic regression models with adjustment for source study, study site, race, education, reference age, first-degree breast cancer family history, age at menarche, number of completed pregnancies, lifetime recreational physical activity, alcohol intake, cigarette smoking status, and oral contraceptive use

Similar results were obtained when analyses were restricted to the two source studies with ER/PR/HER2 status determined in the centralized laboratory (for the highest categories of the two BMI measures vs. the lowest categories of both measures: overall OR = 0.52, 95% CI = 0.33–0.81; luminal-like OR = 0.46, 95% CI = 0.25–0.86). When the analyses were restricted to premenopausal invasive breast cancer case-patient participants (excluding the BCIS Study), results again remained similar (for the highest categories of the two BMI measures BMI vs. the lowest categories of both measures: overall OR = 0.58, 95% CI = 0.40–0.84; luminal-like OR = 0.53, 95% CI = 0.33–0.85).

## Discussion

We pooled data from three population-based case-control studies that included white women and African-American women ages 35–64 years. The analyses of premenopausal women showed that BMI at age 18 years was inversely associated with breast cancer risk overall and with risk of each ER/PR/HER2-defined subtype of breast cancer. These inverse associations did not differ by race. We found no strong evidence of any inverse association between recent BMI and risk of premenopausal breast cancer except when we analyzed the joint effects of BMI at age 18 years and recent BMI, which showed marked inverse associations (range 46–54% decreases in risk) for breast cancer overall and for luminal-like subtype comparing premenopausal women in the highest categories of the two BMI measures (≥ 25 kg/m^2^ at age 18 years and ≥ 30 kg/m^2^ for recent BMI) with women in the lowest categories of the two BMI measures (< 20 kg/m^2^ at age 18 years and < 25 kg/m^2^ for recent BMI). No compelling evidence was observed among postmenopausal women for any associations between BMI at age 18 years or recent BMI and breast cancer overall or ER/PR/HER2-defined subtypes, except for a 1.5-fold increased risk of luminal-like subtype associated with recent BMI among postmenopausal women who had never used MHT.

The Nurses’ Health Study [25], consisting of both premenopausal women and postmenopausal women, showed that BMI at age 18 years was inversely associated with risk of all subtypes defined according to ER/PR/HER2, cytokeratin 5/6, and epidermal growth factor receptor; however, results were not presented separately for premenopausal women and postmenopausal women (≥ 27 vs. < 20 kg/m^2^, luminal A subtype hazard ratio (HR) = 0.5, 95% CI = 0.4–0.8; luminal B subtype HR = 0.7, 95% CI = 0.3–1.4; HER2 HR = 0.6, 95% CI = 0.2–1.7; basal subtype HR = 0.4, 95% CI = 0.1–1.1). Our results also showed that BMI at age 18 years was inversely associated with all tumor marker subtypes and additionally suggested that the inverse association was stronger among premenopausal women than among postmenopausal women. The AMBER Consortium [5] presented results separately for premenopausal and postmenopausal women, reporting that, among premenopausal women, BMI at age 18 years was inversely associated with risk of ER+ breast cancer but not with risk of ER– breast cancer or TNBC (≥ 30 vs. 20–24.9 kg/m^2^, ER+ OR = 0.65, 95% CI = 0.42–1.01; ER– OR = 1.00, 95% CI = 0.63–1.58; TNBC OR = 1.08, 95% CI = 0.59–1.98). However, among postmenopausal women in the AMBER Consortium Study, it was inversely associated with the risk of all subtypes of breast cancer (≥30 vs. 20–24.9 kg/m^2^, ER+ OR = 0.62, 95% CI = 0.38–1.01; ER– OR = 0.78, 95% CI = 0.44–1.41; TNBC OR = 0.68, TNBC 95% CI = 0.29–1.56). The Women’s Health Initiative Cohort Study [26], which included only postmenopausal women, demonstrated that BMI at age 18 years was associated with a 17% reduced risk of ER+ breast cancer, but was not associated with TNBC (≥22.42 vs. < 19.33 kg/m^2^, ER+ OR = 0.83, 95% CI = 0.69–0.98; TNBC OR = 0.94, 95% CI = 0.56–1.56). Based on our pooled results and those of earlier studies, no conclusion can be drawn as to whether the impact of BMI at age 18 years varies by tumor subtype, but at least, all studies support that BMI at age 18 years is inversely associated with risk of breast cancer.

Many previous epidemiologic studies showed that recent BMI was inversely associated with premenopausal women’s risk of breast cancer overall and hormone receptor positive breast cancer, but not with hormone receptor negative breast cancer or TNBC [3, 5]; however, not many of these studies adjusted for BMI in late adolescence in their analyses. Our models assessing effects of recent BMI adjusting for BMI at age 18 years showed only weak evidence of an inverse association between recent BMI and luminal-like subtype; for example, we found a 20% reduced risk of luminal-like subtype associated with recent obesity in premenopausal women. In sensitivity analyses, we removed the variable, BMI at age 18 years, from our models (i.e., no adjustment for BMI at age 18 years) and found that recent obesity was associated with a 29% reduced risk of luminal-like subtype, but was not associated with TNBC or HER2-enriched subtype (≥ 30 vs. < 25 kg/m^2^, luminal-like OR = 0.71, 95% CI = 0.55–0.91; TNBC OR = 0.96, 95% CI = 0.67–1.38; HER2-enriched OR =1.02, 95% CI = 0.60–1.73; per 5 kg/m^2^ increase, luminal-like OR = 0.90, 95% CI = 0.83–0.98; TNBC OR = 1.00, 95% CI = 0.89–1.12; HER2-enriched OR = 1.02, 95% CI = 0.86–1.20, *P* for homogeneity of slope coefficients per 5 kg/m^2^ increase = 0.12, results not shown). Comparing the models with adjustment to those without adjustment for BMI at age 18 years, we note that the magnitude of the protective effect of recent obesity on premenopausal luminal-like cancer was reduced from 29% to 20%. This could explain why we did not observe strong evidence of a negative association between recent BMI and luminal-like subtype of breast cancer in premenopausal women. Nevertheless, we observed clear inverse associations of all ER/PR/HER2-subtypes when assessing BMI at age 18 years after adjusting for recent BMI. Therefore, our data provide evidence suggesting that BMI in late adolescence plays a more important role than recent BMI in premenopausal breast cancer development.

Although our data did not show strong evidence of an inverse association between recent BMI and risk of premenopausal breast cancer, our analyses of the joint effects of BMI at age 18 years and recent BMI in premenopausal women showed the greatest risk reductions for breast cancer overall (46% reduction) and for luminal-like subtype (54% reduction) when contrasting the highest categories of the two variables with the lowest categories. These findings suggest that the reduced risk of premenopausal breast cancer is maximized in women who are consistently overweight or obese during the premenopausal period. Moreover, our results showing a greater reduction in risk for luminal-like subtype than for breast cancer overall associated with recent BMI and the lack of differences in risk reduction across tumor subtypes associated with BMI at age 18 years suggests that recent BMI is more important in determining risk of luminal-like subtype. Yet, results from the AMBER Consortium showing a lack of any impact of the joint effects of BMI at age 18 years and recent BMI on premenopausal ER+ breast cancer risk differ from our results [5]. Thus, further research is needed to clarify such inconsistency.

It has been well-documented that estrogen and progesterone play important roles in breast tumorigenesis [27–29]. A possible mechanism, the suppression of ovarian function resulting in fewer ovulatory menstrual cycles and lower levels of circulating ovarian hormones, that may occur among overweight or obese women [30, 31], could explain the observed inverse associations of BMI at age 18 years and recent BMI with premenopausal breast cancer.

As women pass through menopause, the beneficial effect of obesity on breast cancer risk is replaced by an adverse effect, possibly due to the fact that an important source of estrogen at this time comes from peripheral adipose tissue, where androstenedione is aromatized and converted to estrogen [32, 33]. It has been unclear how long it takes for this transition to occur where BMI changes from a protective factor to a risk factor for breast cancer. Based on the estimates of Pike et al. [34], it takes a decade for a BMI of 30 kg/m^2^ in a premenopausal woman (at age 50 years, relative risk of 0.75) to become a risk factor (relative risk of 1.20 at age 62 years). MacInnis et al. [35] found that in the Melbourne Collaborative Cohort Study, BMI was not associated with risk of breast cancer in women who were postmenopausal for less than 15 years (per 5 kg/m^2^ increase, HR = 0.98, 95% CI = 0.82–1.18), but was associated with a 26% increased risk (per 5 kg/m^2^ increase, HR = 1.26, 95% CI = 1.08–1.46) in women who were postmenopausal for 15 years or more, supporting the notion that prolonged exposure to the proliferative effects of elevated circulating estrogens from adipose tissue is needed. In our pooled data, the upper age limit of participants was 64 years, the average age at reference date for postmenopausal women was 54 years, and only 9% of the postmenopausal women were above age 62 years. Such under-representation of older women might, at least partly, explain why we did not observe consistent positive associations between recent BMI and breast cancer overall or the luminal-like subtype in postmenopausal women.

Our data did show that recent obesity was associated with a 1.5-fold increased risk of luminal-like subtype in postmenopausal women who had never used MHT, but not in those who had used MHT. This could be because the conversion of androstenedione to estrogen in peripheral adipose tissue is negligible when exogenous hormones artificially elevate the amount of circulating estrogens to a comparable level in both lean and obese women [36].

Our pooled analysis has several strengths, including its size, especially the large number of case-patient participants with incident TNBC. Furthermore, the information on body size measures and covariates used in this analysis was collected by trained staff who administrated standardized, in-person interviews using structured questionnaires, which were nearly identical across the three source studies. To our knowledge, we are the first to report results on the associations of both early adulthood and recent BMI with ER/PR/HER2-defined subtypes of breast cancer according to both menopausal status and race.

Several limitations of the current study should be considered. First, our two BMI measures, BMI at age 18 years and recent BMI (representing BMI 5 years prior to reference date), are based on self-reported measures of weight and height. We cannot exclude the possibility that some women may have misreported their weight or height, which could result in the misclassification of BMI. This classification could differ between case-patient participants and control participants, but it is unlikely to differ across ER/PR/HER2-defined case-patient participants. Second, 29% of our case-patient participants had missing data on both ER and PR or on HER2. We compared the distribution of BMI at age 18 years and recent BMI between case-patient participants with known ER/PR/HER2-defined subtypes and those in the undefined group. No statistically significant differences in distribution were detected. Third, ER/PR/HER2 status in two of our source studies [6, 7] was determined in the same laboratory, using the same methods, whereas, in the third source study [8], the information on ER/PR/HER2 status was collected from the LACSP. Using the Women’s CARE Study, we conducted a validation study, which showed that results for the association between reproductive factors and risk of ER/PR subtypes of breast cancer were similar regardless of whether the source of ER/PR information was LACSP or a single centralized laboratory [37]. At that time we were unable to validate HER2 status because the Women’s CARE Study cases had been diagnosed before HER2 data were available in SEER registry records. In addition, when we repeated our analyses with only two of our source studies with ER/PR/HER2 status from the centralized laboratory, the major results were similar (data shown in Table 4). Fourth, the centralized laboratory used IHC to assess HER2 protein overexpression in the Women’s CARE Study and the Women’s BCIS Study, and did not validate results using fluorescent in situ hybridization (FISH). Based on previous validation results from the same pathology laboratory, 7.4% of breast cancers with *HER2* gene amplification in FISH analysis were false negative by 10H8-IHC (scored as 0 or 1+) and 9.7% of breast cancers without *HER2* gene amplification in FISH analysis were false positive [18]. These misclassifications are unlikely to differ by BMI category, but could cause bias towards the null for testing heterogeneity across subtypes, such as TNBC versus HER2-enriched subtype. Fifth, case-patient participants involved in this analysis included those diagnosed with first primary histologically confirmed invasive or in situ breast cancer. Because data are inconsistent regarding whether the BMI in situ breast cancer association is similar to that of BMI with invasive breast cancer [38, 39], we excluded the in situ breast cancer cases and repeated our analyses; we found that the major results based on invasive breast cancers only were similar to those based on both invasive cases and in situ cases (data shown in Table 4).

## Conclusions

Our findings indicate that high BMI near the end of adolescence decreases risk of all ER/PR/HER2-defined subtypes of premenopausal breast cancer and also suggest that this benefit could be maximized among premenopausal women who consistently have high BMI during their premenopausal years.

## Abbreviations

AMBER: African-American Breast Cancer Epidemiology and Risk
BCIS: Breast carcinoma in situ
BMI: Body mass index
CAP: College of American Pathologists
CARE: Contraceptive and Reproductive Experiences
CI: Confidence interval
CLIA: Centralized clinical laboratory improvement act
ER: Estrogen receptor
FISH: Fluorescent in situ hybridization
HER2: Human epidermal growth factor receptor 2
ICD-O: International Classification of Diseases for Oncology
IHC: Immunohistochemistry
LACSP: Los Angeles County Cancer Surveillance Program
LCIS: Lobular carcinoma in situ
LIFE: Learning the Influence of Family and Environment
MHT: Menopausal hormone therapy
OR: Odds ratio
PR: Progesterone receptor
SEER: Surveillance, Epidemiology and End Results
TNBC: Triple negative breast cancer
USC: University of Southern California
